# *Callitrichine gammaherpesvirus* 3 and *Human alphaherpesvirus* 1 in New World Primate negative for yellow fever virus in Rio de Janeiro, Brazil

**DOI:** 10.1590/0074-02760210258

**Published:** 2022-04-11

**Authors:** Flávia Freitas de Oliveira Bonfim, Maria Angélica Monteiro de Mello Mares-Guia, Marco Aurélio Horta, Marcia Chame, Amanda de Oliveira Lopes, Rafael Santos, Carlos Alexandre Rey Matias, Marcelo Alves Pinto, Ana Maria Bispo de Filippis, Vanessa Salete de Paula

**Affiliations:** 1 Fundação Oswaldo Cruz-Fiocruz, Instituto Oswaldo Cruz, Laboratório de Virologia Molecular, Rio de Janeiro, RJ, Brasil; 2 Fundação Oswaldo Cruz-Fiocruz, Instituto Oswaldo Cruz, Laboratório de Flavivírus Molecular, Rio de Janeiro, RJ, Brasil; 3 Fundação Oswaldo Cruz-Fiocruz, Plataforma Institucional de Biodiversidade e Saúde Silvestre, Rio de Janeiro, RJ, Brasil; 4 Fundação Oswaldo Cruz-Fiocruz, Instituto Oswaldo Cruz, Laboratório de Doenças Parasitárias, Rio de Janeiro, RJ, Brasil; 5 Universidade Federal Rural do Rio de Janeiro, Instituto de Veterinária, Departamento de Epidemiologia e Saúde Pública, Rio de Janeiro, RJ, Brasil; 6 Fundação Oswaldo Cruz-Fiocruz, Instituto Oswaldo Cruz, Laboratório de Desenvolvimento Tecnológico em Virologia, Rio de Janeiro, RJ, Brasil

**Keywords:** *Callitrichine gammaherpesvirus* 3, *Human alphaherpesvirus* 1, non-human primates

## Abstract

**BACKGROUND:**

Herpesvirus transmission between humans and non-human primate (NHP) can occur through contact scratches with lesions, infected saliva, and mainly through contaminated food. Therefore, cross-infection can lead to severe illness or even death for both the animal and human. In 2017, during the yellow fever (YF) outbreak in Brazil, species of the New World Primates (NWP) from Rio de Janeiro state, tested negative for yellow fever virus (YFV) detection.

**OBJECTIVES:**

To evaluate herpesvirus in the population NWP in Rio de Janeiro.

**METHODS:**

To investigate, liver samples of 283 NWP, from several regions of the state of Rio de Janeiro, were tested for the herpesvirus family using a *Pan*-polymerase chain reaction (*Pan*-PCR) and sequencing.

**FINDINGS:**

34.6% (98/283) tested positive for at least one herpesvirus; 29.3% (83/283) tested positive to *Human alphaherpesvirus* 1 (HSV-1), this virus from humans can be lethal to New World monkey; 13% (37/283) were detected *Callitrichine gammaherpesvirus* 3 (CalHV-3), responsible for lymphoproliferative disease that can be fatal in NWP. In addition, CalHV-3 / HSV-1 co-infection was in 11.6% (33/283) of the samples.

**MAIN CONCLUSIONS:**

*Pan*-herpesvirus was useful to identify species-specific herpesviruses and virus from human that can infect animals. Furthermore, during an outbreak of YF other infections should be monitored.

Human action on ecosystems can cause irreversible environmental changes, leading to imbalances in biological systems, species extinction, increasing interaction between wildlife and humans that generates the spillover and the emergence and resurgence of a wide variety of infectious and zoonotic agents.^1^ ^,^ ^2^ ^,^ ^3^ Brazil has a wide variety of naturally preserved ecosystems and biodiversity, but harmful human actions have modified the fauna and flora, such as conserved landscapes coexisting with deforestation and urbanised areas.^4^ Brazil has the largest number of native and exotic primate species and the largest number of endangered primates in the world, about 38% of them are threatened and 48% are declining in Brazil.^5^ New World Primate (NWP) are vulnerable to the introduction of exotic pathogens as well as human pathogens.^6^

NWP and humans host a variety of herpesviruses.^7^ These viruses usually cause asymptomatic infections in their natural host but they are associated with severe disease when transmitted to different species. Herpesvirus interspecies transmission carries a high zoonotic risk and can result in fatal human or monkey diseases. Herpesvirus transmission depends on the intimate contact between human and NHP through contaminated respiratory droplets, saliva or food between a susceptible individual and individual who is excreting the virus as observed with NWP.^8^

Neotropical primate is susceptible to *Human alphaherpesvirus* 1 (HSV-1) infections and disease.^9^ The course of the disease can be severe and may lead to death. Clinical manifestations in NWP of HSV-1 may be similar to primary manifestations in humans characterised by oral ulcerative, vesicular lesions, periocular, nasal, conjunctivitis, apathy, anorexia and ataxia, but in most cases, it is fatal. In contrast, HVS-1 infections of Old World Primate remain localised and cause only mild mucocutaneous lesions.^10^ ^,^ ^11^ HSV-1 lesions were reported in naturally infected Neotropical Primate,^12^ ^,^ ^13^ in experimentally infected marmosets (*Callithrix* spp., *Saguinu*s spp.).^14^ There are spontaneous HSV-1 infection fatal cases in marmosets following contact with humans with an HSV-1 injury and asymptomatic, are constantly reported.^15^ ^,^ ^16^ ^,^ ^17^

Epstein-Barr virus (EBV) is *Lymphocryptovirus* (LCV) that can be asymptomatic or symptomatic. EBV is associated with a variety of human diseases, including infectious mononucleosis, B-cell malignancies, epithelial cell malignancies and oncogenic linfocryptovirus.^18^ *Callitrichine gammherpesvirus* 3 (CalHV-3), homologous to Epstein Barr Virus (EBV), can infect NWP.^19^ In 2002, CalHV-3 was identified as a member of lymphocriptovirus (LCV).^20^ ^,^ ^21^ That can be fatal in marmoset, the natural host. The natural host to EBV simian homologues may provide an important animal model to study the pathogenesis, oncology, and evolution of Lymphocryptoviruses for a better understanding of EBV cell and host.^22^ There is a residual zoonotic potential of viruses homologous to Epstein Barr from animals to humans, being able to cross the host barrier and adapt to new hosts.^23^ ^,^ ^24^

Yellow fever (YF) epizootics affected several species of NHP,^25^ ^,^ ^26^ ^,^ ^27^ reaching Central and South America.^27^ ^,^ ^28^ ^,^ ^29^ During the YF outbreak in Southern Brazil, the state of Rio de Janeiro notified epizootic diseases involving NHP.^30^ That are sentinels for the detection of human cases of the disease.^31^ In Brazil, since 1999, the monitoring of infection in NWP generates indicators of transmission in relation to the wild and human cycle.^32^ Among all NWP found dead, 78% tested negative for yellow fever virus (YFV),^32^ though they may have been infected by some other pathogen.^33^ ^,^ ^34^ Therefore, in 2017, the circulation of herpesvirus was investigated within the population of NHPs that died, suspected of YF in the state of Rio de Janeiro, Brazil.

## SUBJECTS AND METHODS

*Animals* - During the YF outbreak that occurred in 2017, the NWP found dead in several regions and municipalities of the state of Rio de Janeiro were analysed to YF at the Flavivirus Laboratory (LABFLA) of the Oswaldo Cruz Foundation (Fiocruz) in Rio de Janeiro, within the surveillance program of YF. Table I shows the primate genus or species of the NWP analysed in this study. The LABFLA is a laboratory from Brazilian Ministry of Health Regional Reference Laboratory for Arboviruses.^32^

**TABLE I:** Distribution of non-human primates according with genus and sex

Genus	N	Sex		
		Female (%)	Male (%)	Unknown (%)
*Alouatta* spp.	10	2 (20)	7 (70)	1 (10)
*Leontopithecus* spp	1	0	1(100)	0
*Sapajus* spp.	7	1 (14.3)	6 (85.7)	0
*Callithrix* spp.	265	125 (47.2)	121 (45.0)	19 (7.1)
Total	283	130 (45.9)	136 (48.0)	17 (6.0)

In this study, 283 liver samples that tested negative to YF PCR were referred for herpesvirus diagnosis at Molecular Virology Laboratory (LVM), Fiocruz. Each animal was identified according to species, sex, location and age. As these are samples of convenience received during the epidemiological investigation of YF outbreaks, conducted by the Health Surveillance Bodies of the Brazilian Ministry of Health, and there is no manipulation of animals, submission to the ethics and animal use committee and the license of Instituto Chico Mendes de Conservação da Biodiversidade (ICMBio‐SISBio) was not necessary. This research was funded by the Ministry of Health of Brazil in emergency public health response to the outbreak of YF in the NWP. The samples were provided for surveillance and research purposes pursuant to Resolution 2,998,362 IOC/Fiocruz. The research design approved by the appropriate ethical review board.

*Ethical approval* - The study was approved by the Brazilian Ministry of Health and the samples were obtained during the YF outbreak. Samples were provided for surveillance and research purposes within the terms of Resolution 2.998.362 IOC/Fiocruz. The study design was approved by the appropriate ethics review board.

*Extraction of DNA for molecular diagnostics* - Serum samples obtained from NHP were processed in a biosafety level 3 (BSL3) environment and stored at -70ºC until tested. Approximately 30 mg of liver tissue were disrupted in 600 μL of lysis buffer; an aliquot of 115 μL of the lysate was then mixed with 20 μL of bead mix plus and 65 μL of 100% isopropanol. Nucleic acids were extracted using MagMAX^TM^ Pathogen RNA/DNA kit (Life Technologies, Carlsbad CA, USA) in accordance with the manufacturer’s instructions. The DNA was stored at -70ºC until processing.

*Pan-herpesvirus* - Herpesvirus detection was performed using the *Pan*-herpesvirus polymerase chain reaction described by Ehlers et al.,^35^ target Dpol region. This reaction simultaneously detects virus from the *Herpesviridae* family that infects humans and animals.

Three primers, degenerate and with deoxyinosine (deg/dI), were used in the first-round PCR: 285s DFA (5’GAYTTYGCIAGYYTITAYCC3’), 285s ILK (5’ TCCTGGACAAGCAGCARIYSGCIMTIAA 3’) and 285as KG1 (5’ GTCTTGCTCACCAGITCIACICCYTT 3’). Two primers were used in the second-round as follows: 286sTGV (5’ TGTAACTCGGTGTAYGGITTYACIGGIGT 3’) and 286-as IYG (5’ CACAGAGTCCGTRTCICCRTAIAT 3’. Each PCR mix contained a total volume of 25 µL with 2.5 µL of each primer (forward/reverse 10 µM), 1.25 µL dimethyl sulfoxide 100x (Life Technologies, California, USA), and 2 µL MgCl_2_ (25 mM), 2.5 µL of 10X PCR buffer with 15 mM MgCl_2_ (Applied Biosystems GmbH, Darmstadt, Germany) and 1 µL of deoxynucleoside triphosphate (10 mM), 0.25 µL of DNA polymerase AmpliTaq Gold (5U). The first - and second - round PCRs were performed for 45 and 35 cycles, respectively. At the beginning, with the activation of the polymerase of 12 min at 95ºC, the reactions were subjected to 20 s of denaturation at 95ºC, 30 s of annealing at 46ºC and 30 s of strand extension at 72ºC, followed by a final extension step at 72ºC for 10 min.

The amplicons were analysed by electrophoresis gel in 1.5% agarose gel with ethidium bromide (0.5 μg/mL) (Invitrogen, USA), and observed under UV light (Benchtop UV transilluminator Uplan, CA, USA).

To determine which type of herpesvirus, direct nucleotide sequencing was performed in both directions, the products of the second-round PCRs (3.2 pmol concentration) were sequenced using reagents and protocols of the ABI Kit BigDye Terminator version 3.1 cycle sequencing kit (Applied Biosystems, Foster City, CA, USA) and ABI 3730xl automated sequencers (Applied Biosystems, Foster City, CA, USA).

The sequences were analysed in the 7.2.5 BioEdit program and compared with others deposited in GenBank, using BLAST (Basic Local Alignment Search Tool) to identify herpesviruses species that were detected in the Pan-PCR.

*Qualitative PCR for HSV-1 by PCR region gG* - To confirm HSV-1 detection, samples showing positivity in Nested PCR were additionally amplified by a gG region of *Human alphaherpesvirus* 1 PCR. The PCR HSV-1 was performed in a reaction mixture comprising 14.9 μL of water RNAse/DNAse *free* (Gibco, NI, USA), 2.5 μL of 10x PCR *Buffer* I (Applied Biosystems, Foster City, CA, USA), 0.5 μL of dNTP (10 mM) (Invitrogen, CA, USA) 0.5 μL of each oligonucleotide (0.2 μM), 0.75 μL of MgCl₂ (50mM) (Applied Biosytems, USA), and 0.1 μL of *Taq Polymerase Platinum* (5 U) (Thermo Fisher Scientific, Invitrogen, USA) and 1 μL DNA. Specific oligonucleotides were used for viral amplification of the glycoprotein G region as follows: gG F (5’ -GACTCTCCCACCGCCATCAG- 3’) and gG R (5’ -TGTCTTCGGGCGACTGGTCT-3’).^36^ ^,^ ^37^ ^,^ ^38^

Aliquot of HHV-1 strain KOS^39^ with a viral titer of 10^7^ copies/mL from cell culture was diluted from 1 to 10^5^ copies/mL in RNAse/DNAse free water (Gibco, NI, USA) after extraction using the commercial QIAamp^®^ DNA Purification from Blood or Body Fluids kit (QIAgen, Valencia, CA, USA) according to the protocol. After serial dilution, amplification was performed, followed by running on gel electrophoresis. The results acquired from the detection limit were evaluated and the samples from the serial dilution for use as positive controls, then ultrapure water samples were and negative serum used as negative controls.

PCR mix containing reactions of the PCR HSV-1 were performed for 40 cycles. At the beginning, with the activation of the polymerase of 5 min at 96ºC, the reactions were subjected to 45 s of denaturation at 95ºC, 45 s of annealing at 58ºC and 45 s of strand extension at 72ºC, followed by a final extension step at 72ºC for 10 min. The PCR product was analysed by 1.5% agarose gel, with ethidium bromide (0.5 μg/mL) (Invitrogen, USA), and observed with UV light (Benchtop UV transilluminator Uplan, CA, USA). Direct nucleotide sequencing was performed in both directions from products of the PCR HSV-1 gG (3.2 pmol), using reagents and protocols of the ABI Kit BigDye Terminator version 3.1 cycle sequencing kit (Applied Biosystems, Foster City, CA, USA) and ABI 3730xl automated sequencers (Applied Biosystems, Foster City, CA, USA) to identify the *Human alphaherpesvirus* 1 sequence. The sequences found of the reactions were analysed in the 7.2.5 BioEdit program and compared with others deposited in GenBank, using the BLAST (Basic Local Alignment Search Tool).

*Statistical snalysis* - Data obtained from the analysed samples were correlated with age, location, gender, sex and necropsy. Molecular test results were categorised and stored in a database created in Microsoft Office Excel (Microsoft Corporation, USA). The statistical analysis was performed only on genus *Callithrix* spp. due to the reduced number of animals from other genera. The chi-square and t-test were used to compare independent samples. Analysis were performed with R software version 3.5.0. A p-value < 0.05 was considered statistically significant.

*Mapping and distribution of herpesvirus* - The ArcMap 10.5 program was used to create maps with the municipalities of Rio de Janeiro and prevalence of Pan-PCR and *Human Alphaherpesvirus* 1. The cartographic basis for building the map was freely extracted from the Brazilian Institute of Geography and Statistics (IBGE).

## RESULTS

*Distribution of NWP* - Genera *Callithrix* spp., *Alouatta* spp., *Leontopithecus rosalia* and *Sapajus* spp. were observed among 283 NWPs. The majority (93%) belonged to genus *Callithrix* spp. The total of animals tested in this study was 283, however, not all animals had all information available. Of the total NWP 45.5% (128/283) were female and 47.7% (135/283) were male. Among these, 38.1% (108/283) suffered some type of (see Tables I-II).

**TABLE II:** Distribution, prevalence of Pan-herpesvirus, *Human alphaherpesvirus* 1 (HSV-1), HSV-1/*Callitrichine gammaherpesvirus* 3 (CalHV-3) co-infection and Chi-square test values for samples of non-human primates (NHP) from the state of Rio de Janeiro

		*PAN-*Herpesvirus		HSV-1		HSV-1/CalHV-3
	N (%)	Pos* (%)	p**	*Pos (%)	p**	*Pos (%) p**
Genus			0.58		<0.01	0.13
*Callithrix* spp.	265 (93,6)	93 (35.1)		81 (28.6)		32 (11.3)
*Sapajus* spp.	7 (2.4)	1 (14.3)		1 (0.35)		1 (0.3)
*Alouatta* spp.	10 (3.6)	4 (40.0)		1 (0.35)		-
*Leontopithecus* spp.	1 (0.4)	0 (0.0)		-		-
Total	283	98 (34.6)		83(29.3)		33 (11,6)
Sex			0.16		0.86	0.90
Female	130 (45.0)	52 (40.0)		44 (16.6)		18(6.7)
Male	135 (51.0)	42 (31.1)		35 (13.2)		13 (5.0)
Total	265^&^	94(35.4)		79(29.8)		31 (11.6)
Necropsy			0.57		<0.05	0.08
Trauma	108 (96.4)	36 (33.3)		28 (25.0)		7(6.2)
Intoxication	2 (1.8)	1 (50.0)		1 (0.8)		-
Heartworm disease	1 (0.9)	0 (0.00)		-		-
Jaudice	1 (0.9)	1 (100.0)		-		-
Total	112^&^	38		29 (25.9)		7(6.2)
Collection site			0.66		0.15	0.57
Rio de Janeiro	108 (38.1)	39 (36.1)		33 (17.0)		13(66.6)
Niterói	44 (15.5)	19 (43.2)		17 (8.7)		7(3.5)
Petrópolis	28 (9.8)	11 (39.3)		9 (4.6)		4(2.0)
Duque de Caxias	8 (2.8)	1 (12.5)		1 (0.5)		1(0.5)
Volta Redonda	7 (2.4)	0 (0.00)		-		-
Angra dos Reis	9 (3.1)	3		3		1
Araruama	1(0.3)	1		1		1
Areal	2 (0.7)	-		-		-
Bom Jardim	1(0.3)	-		-		-
Búzios	1(0.3)	-		-		-
Cachoeira de Macacu	3(1.0)	2		2		1
Campo dos Afonsos	1(0.3)	-		-		-
Campo dos Goytacazes	2(0.7)	1		1		1
Duas Barras	1(0.3)	-		-		-
Guapimirim	2(0.7)	1		1		-
Itaboraí	2(0.7)	-		-		-
Itaguaí	4(1.4)	2		2		-
Itatiaia	1(0.3)	1		1		-
Macuco	1(0.3)	-		-		-
Magé	5(1.7)	3		3		2
Mangaratiba	2(0.7)	1		1		1
Maricá	5(1.7)	2		2		2
Miracema	1(0.3)	-		-		-
Nova Friburgo	5(1.7)	2		2		-
Nova Iguaçu	4(1.4)	1		1		-
Paracambi	3(1.0)	2		1		-
Parati	3(1.0)	-		-		-
Paty do Alferes	1(0.3)	1		-		-
Pinheiral	1(0.3)	-		-		-
Piraí	2(0.7)	-		-		-
Queimados	3(1.0)	1		1		-
Resende	1(0.3)	-		-		-
Rio Claro	2(0.7)	1		1		-
Rio Comprido	1(0.3)	-		-		-
Rio das Flores	1(0.3)	-		-		-
Rio das Ostras	2(0.7)	-		-		-
São Gonçalo	5(1.7)	1		1		-
São João de Meriti	1(0.3)	-		-		-
São José de Ubá	1(0.3)	-		-		-
São Pedro da Aldeia	1(0.3)	1		-		-
Saquarema	2(0.7)	-		-		-
Silva Jardim	2(0.7)	1		-		-
Teresópolis	3(1.0)	-		-		-
Vassouras	3(1.0)	-		-		-
Xerém	1(0.3)	-		-		-
Total	283	98 (34.6)		83(29.3)		33(11.6)
Age group *Callithrix* (Months)			0.0031		0.27	0.28
Infant (0-5)	33 (12.1)	9 (3.3)		6 (2.2)		3 (1.1)
Young (5.1-10)	30 (11.0)	1 (0.3)		1 (0.3)		-
Subadult (10,1-15)	18 (6.6)	6 (2.2)		6 (2.2)		3(1.1)
Adult (>16)	190(70.1)	76(28.0)		64 (23.6)		28(10.3)
Total	271 ^&^	92(34.0)		75(27.6)		34(12.5)

*: positive; **: p-value; -: zero; &: the total number is according information available.

*Detection of herpesvirus through pan-herpesviruses* - Among the 283 samples analysed, 34.6% (98/283) were positive for at least one herpesvirus. In theses NHP, 94.9% (93/98) were from genus *Callithrix* spp., 4.08% (4/98) from *Alouatta* spp.; 1.02% (1/98) from *Sapajus* spp. (see Table II). The variables genus, sex, necropsy and collection site were not considered statistically significant with p < 0.05, however the age group was statistically significant with p-value at 0.0031.

The highest prevalence for this herpesvirus was found in urban areas, including the cities of Rio de Janeiro, Niteroi, Petropolis, Mage and Angra dos Reis and the other municipalities, according to Table II.

*Detection of Human alphaherpesvirus 1* - As the majority of samples were from urban areas, where contact between human and NHP happens in the cities of Rio de Janeiro, Niteroi, Petropolis, Mage and Angra dos Reis and the other municipalities, according to Table II and Figure, all positive samples in *Pan*-PCR were also confirmed by a specific PCR to HSV-1, target gG region. The Figure shows prevalence of number of cases in relation to a total of 283 NHP tested for HSV-1. Of the 98 *Pan*-herpesvirus positive samples, 84.7% (83/98) tested positive to HSV-1 DNA (see Table II). The samples analysed showed that 29.3% (83/283) tested positive to HSV-1. The variables genus, sex and collection site were not considered statistically significant with p < 0.05, however the age group and genus was statistically significant with p-value at 0.0031 and p < 0.05, respectively. The genus *Callithrix* shows a significant difference in mean age between negative (14,2) and positive (5,6) animals when diagnosed by the HSV-1gG (t = 4.43; p<0.001) method. And, *Pan*-herpesvirus PCR, the result was similar with an average of 14,8 for negatives and 5,6 positives (t = 4.68; p < 0,001).

**Figure:**
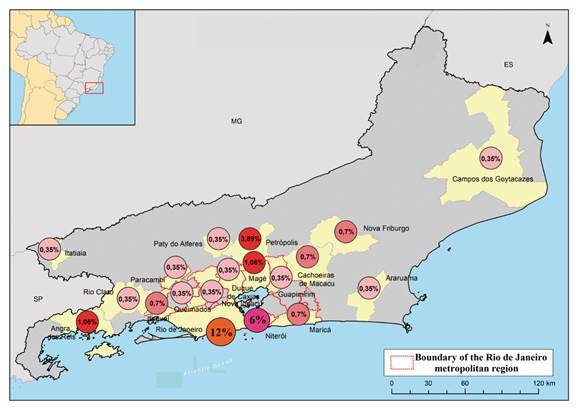
Distribution of prevalence of *Human alphaherpesvirus* 1 (HSV-1) in non-human primates detected in municipalities in Rio de Janeiro state, Brazil. The Figure shows prevalence of number of cases in relation to a total of 283 non-human primate (NHP) tested for HSV-1.

The samples analysed showed that 13% (37/283) were detected with CalHV-3. In addition, CalHV-3/HSV-1 co-infection was observed in 11.6% (33/283) of the samples (See Table II).

## DISCUSSION

NWP are highly susceptible to herpesvirus infections, which are easily disseminated among group members in the wild.^9^ ^,^ ^39^ ^,^ ^40^ Among the 354 liver samples analysed from NHP during a YF epizootic, 20% (71/354) were positive for YFV^32^ and 80% (283) that were negative to YFV was sent to research for herpesvirus. In the last decades, YFV infections have been prevalent in endemic areas in Brazil, affecting human and NWP populations. Monitoring of the NHP infection started in 1999 and reports of epizootic diseases are considered important indicators of viral transmission, particularly in relation to the sylvatic cycle. However, in 2017, during YF epizootic, in a large percentage of NHP investigated, YF-RNA was not detected. For this reason, we conducted the investigation of herpesvirus in negative NWP negative for YF.

In this study, with a total of 283 liver samples, were positive for at least one type of herpesvirus 34.6% (98/283). *Callitrichine gammaherpesvirus* 3 (13%, 37/283) infection was identified in NHP, a virus similar to *Human gammaherpesvirus* 4 (Epstein Barr), which can cause lymphoproliferative disease that infects simian. Previous studies show that EBV-related herpesviruses are endemic in NWP families with a prevalence of more than 50%.^19^ ^,^ ^21^ ^,^ ^32^ ^,^ ^41^ Generally, gammaherpesviruses do not cause serious disease in the primary infection of their natural host and it may remain latent throughout the life of the animal, but due to some factors, such as low immune system or transmission to divergent species, it can cause fatal cases. In turn, it can induce viral lymphoproliferation and rapidly become a malignant lymphoma or mononucleosis, leading to the death of the animal. It is responsible for lymphoproliferative disease that can be fatal in NWP. In addition, studies have been reported that latently infected animals can develop the disease only when immunocompromised, either by research-related manipulation, concomitant disease or co-infection.^14^ ^,^ ^42^ ^-^ ^51^ In 2001, Young-Gyo Cho’s team at Harvard Medical School isolated B-cell CalHV-3 from *Callithrix jacchus* lymphomas, indicating that persistent EBV-like virus infection is prevalent in *Callitrhix* as well as in squirrel monkeys, which is a NWP species, and that CalHV-3 infection in marmosets may also provide an animal model for EBV pathogenesis and associated neoplasia in humans.^19^

*Callithrix jacchus* species, fully inserted in the Atlantic Forest in the state of Rio de Janeiro, is one of the largest species of introduced Neotropical primate, whose original habitat is northeastern Brazil, currently adapted to this habitat, peri-urban and the urban environment, such as forest parks and rural areas. *C. penicilatta* species, also introduced in Rio de Janeiro forming hybridises with *C. jacchus.* Through closer contact with humans, in search of food or through the intrusion of their natural habitat. The HSV-1 virus can be carried by human saliva in NHP food waste, and this can be fatal.^8^ ^,^ ^9^ ^,^ ^10^ ^,^ ^15^ ^,^ ^39^ ^,^ ^52^ ^-^ ^55^ Pathogenicity in NWP HSV-1 is similar to the primary manifestations in humans, characterised by ulcerative vesicular lesions, oral, periocular, nasal, conjunctivitis, apathy, anorexia and ataxia, but it can be fatal in these animals. NWP are more susceptible to HHV-1 infections and diseases, the course of the disease is severe, leading to death in most cases.^10^ ^,^ ^11^

The presence of HSV-1 was evaluated by two region of HSV-1 and the prevalence of HSV-1 was 29.3% (83/283) among NHP tested. In a study conducted in Thailand, a similar prevalence of 28.2%, was found in gibbons.^56^ The high detection of HSV-1 DNA found in our study can be explained by the close contact between humans and *Callithrix* spp. or accounted for by monkey-monkey transmission following an initial introduction from human contact. Currently in Brazil, the genus *Callithrix* spp. is the NWP that has the closest contact with humans, and they, in turn, live in large groups sharing food, for example, thereby facilitating the transmission of the infectious agent between NHP and between the distinct genres of NHP.^57^ ^,^ ^58^ As humans are the natural hosts and reservoir of HSV-1, the transmission can happen easily and lethal,^9^ ^,^ ^10^ ^,^ ^11^ since New World monkeys are susceptible to HSV-1 infections and disease. This virus from humans can be lethal to NWP. In non-human primates, the course of the disease is severe, leading to death in most cases.^59^ ^,^ ^60^ High mortality through HSV-1 indicates the need for prophylactic strategies, epidemiology surveillance, biological conservation, control of the spread of infection among individuals in the same group to prevent transmission and effective treatment in infected animals.^52^ NWP are vulnerable to HSV-1 infection and it leads to neural disorders making these animals, which are arboreal, vulnerable to tree falls and causing polytrauma. According Table II, shows the necroscopy variable in which 96.4% (108/112) of the cases analysed by the veterinarians were trauma of unknown origin.

The highest prevalence of herpesvirus was found in *Callithrix* spp., 94.9% (93/98), followed by *Alouatta* with 4.1% (4/98) and only 1.02% in *Sapajus* (1/98). According to Chico Mendes Institute for Biodiversity Conservation (ICMBio), Brazil is the country with the largest number of known primates and about 40% of primate species are endangered. In this study, there are two species on the list of threatened, according to ICMBio, *Alouatta* spp. and *Leontopithecus* spp., which in turn increases the level of vulnerability due to the fact that other invasive and exotic primate species such as *Callithrix* spp. compete for the habitat.^60^ ^,^ ^61^ And these ICMBio data corroborate the study data, in which there is a discrepant difference between the threatened species with about 11 NWP and the invasive and exotic *Callithrix* spp. with 265 NWP, it is important transmitter of infectious disease for native primates populations in the regions.

In this study, it was described for the first the co-infection with the subfamily *Gammaherpesvirinae* and *Alphaherpesvinae* viruses in NWP. The HSV-1/CalHV-3 co-infection could contribute to the death of animals. Unfortunately, the prevalence of antibodies was not detected because the serum samples were not available. The age group results were statistically significant of detection of Pan-herpervirus DNA in Callitrichidae family (p = 0.0031).

Although there is no statistical correlation, and considering the period of life, we supposed that adults could be more exposed by foraging for food or by transmission within your group.

In conclusion, *Pan*-herpesvirus was useful to simultaneous detection of herpesvirus infections in NWP and to identify not only species-specific herpesviruses, but also to virus from human that can infect animals. Furthermore, it was useful to warn, during an outbreak of YF, that other infections should be monitored and investigated and finally it can be included as a complementary program of the Ministry of Health, as the NWP are closely linked to men in their physiology and immune system and to highlight the importance of the behavior and the awareness of the population about living and managing with animals in natural, public and forest spaces.
